# Fibroblast Activation Protein-Targeted Radioligand Therapy (FAP RLT) in Gastroenteropancreatic Malignancies: Biological Rationale, Clinical Evidence, and Future Directions

**DOI:** 10.1055/s-0046-1820110

**Published:** 2026-04-11

**Authors:** Deya' Aldeen Sweedat, Saad Ruzzeh, Ahmed Saad Abdlkadir, Serin Moghrabi, Marwah Abdulrahman, Akram Al-Ibraheem

**Affiliations:** 1 Department of Nuclear Medicine, King Hussein Cancer Center (KHCC), Amman, Jordan; 2 Department of Nuclear Medicine, Baghdad Radiotherapy and Nuclear Medicine Hospital, Bab Al-Muadham, Baghdad, Iraq

**Keywords:** gastroenteropancreatic cancers, fibroblast activation protein, FAPI PET/CT, ^177^ Lu-FAP, radioligand therapy, theranostics

## Abstract

Gastroenteropancreatic (GEP) malignancies are a major cause of cancer-related mortality, with many patients presenting at advanced stages where curative options are limited. Outcomes remain suboptimal, particularly beyond first-line therapy, highlighting the need for novel approaches. Fibroblast activation protein (FAP)-targeted radioligand therapy (RLT) has emerged as a biologically rational strategy that exploits the stromal compartment of desmoplastic tumors. FAPI PET/CT enables noninvasive assessment of target expression and supports a theranostic framework for patient selection. This review summarizes current clinical evidence on Lutetium-177 ( ^177^ Lu)-FAP RLT in GEP cancers, including pancreatic, biliary, gastric, and colorectal malignancies. Available data, largely from small and heterogeneous early-phase studies, demonstrate feasibility and acceptable short-term safety, with hematologic toxicity as the main concern. Objective responses are uncommon; however, disease stabilization and symptomatic benefit have been observed in selected patients. Key limitations include variable tumor retention, heterogeneous selection criteria, and the lack of standardized dosimetry and response assessment. Overall, ^177^ Lu-FAP RLT remains investigational but represents a biologically compelling but still clinically unproven biomarker-driven strategy, whose current role is best confined to investigational settings and highly selected patients. Future progress requires optimized ligand design, standardized imaging and dosimetry, and prospective clinical trials to define its clinical role.

## Introduction

Gastroenteropancreatic (GEP) cancers remain a major contributor to global cancer mortality, reflecting the combined burden of common high-incidence tumors such as colorectal and gastric cancer and highly lethal malignancies such as pancreatic and liver. 1 GEP malignancies frequently come to clinical attention at an advanced or metastatic stage, particularly pancreatic and biliary tract cancers, for which a large proportion of patients are unresectable at diagnosis and are therefore managed with palliative-intent systemic therapy rather than curative surgery. 2 3 In advanced gastrointestinal malignancies, systemic treatment is largely based on cytotoxic chemotherapy regimens, with only modest efficacy, particularly beyond the first-line setting. 4 Although targeted therapies and immunotherapy have improved outcomes in selected biomarker-defined subgroups, these benefits are limited to a minority of patients, and durable disease control remains uncommon in later lines. 5 Accordingly, there is a strong clinical rationale to explore alternative approaches, including fibroblast activation protein-targeted radioligand therapy (FAP RLT), in patients who have exhausted standard treatment options. 6 7

Fibroblast activation protein inhibitor positron emission tomography/computed tomography (FAPI PET/CT) has emerged as a promising imaging modality in GEP malignancies by targeting cancer-associated fibroblasts (CAFs) within the tumor stroma, often achieving higher tumor-to-background contrast than Fluorine-18 Fluorodeoxyglucose positron emission tomography/computed tomography ( ^18^ F-FDG PET/CT), particularly in tumors with dense desmoplasia or relatively low FDG avidity. 8 Strong evidence indicates that FAPI PET/CT demonstrates higher pooled sensitivity than FDG PET/CT across several digestive system cancers, with particular advantages in gastric, hepatic, biliary, and pancreatic tumors, and improved detection of nodal and peritoneal disease in colorectal cancer. 9 10 11 12 These features support the theranostic potential of FAPI, with early clinical data suggesting antitumor activity and a favorable safety profile for FAPI-based RLT, although current evidence remains limited and heterogeneous. 7

RLT has already validated “target-and-treat” oncology in other diseases: Lutetium-177( ^177^ Lu)-DOTATATE improved progression-free survival in advanced midgut neuroendocrine tumors (NETTER-1), 13 and ^177^ Lu-PSMA-617 improved both imaging-based progression-free survival and overall survival in metastatic castration-resistant prostate cancer (VISION). 14 These results support expanding RLT beyond tumor-cell–specific targets toward shared microenvironmental components, particularly the tumor stroma.

This review synthesizes the emerging clinical literature on ^177^ Lu-labeled FAP-targeted RLT in GEP malignancies, with emphasis on pancreatic, biliary tract, gastric/gastroesophageal, and colorectal cancers (CRCs), and particular consideration of the therapeutic challenges posed by liver-dominant disease. It examines currently available clinical outcomes, dosimetric findings, and organ-at-risk considerations, especially with respect to bone marrow tolerance and hepatic functional reserve, while also addressing practical aspects of patient selection based on FAP PET imaging and the evolving challenges of response assessment in stromal-targeted therapy. The review was based on a structured search of PubMed and Scopus through February 2026 for clinical studies of FAP-targeted RLT in GEP malignancies, with additional screening of reference lists from relevant articles to maximize completeness.

### Biological Rationale for FAP-Targeting in GEP Cancers

Fibroblast activation protein (FAP) is a membrane-bound serine protease expressed mainly on activated fibroblasts involved in tissue remodeling and on CAFs. In solid tumors, FAP-positive CAFs are a major stromal component and are linked to extracellular matrix remodeling, tumor progression, and adverse prognosis. GEP malignancies, particularly pancreatic ductal adenocarcinoma and cholangiocarcinoma, are characterized by marked desmoplasia, making stromal targeting biologically attractive, especially when tumor-cell targets are heterogeneous or variably expressed. 15 16 17

In GEP cancers, desmoplasia is not only a structural feature but also a contributor to treatment resistance. Dense stromal matrix can impair drug delivery, while CAF-derived signals promote tumor survival, invasion, and aggressive behavior. In pancreatic and biliary tract cancers, CAF-mediated immune exclusion and immunosuppression may further limit response to systemic therapies, including immunotherapy. 18 19 20

RLT with ^177^ Lu emits β particles with a tissue penetration range sufficient to generate a crossfire effect, allowing radiation delivery to neighboring tumor cells even when the radioligand binds primarily to stromal elements rather than malignant cells directly. Because CAFs are distributed throughout the tumor microenvironment, stromal targeting may provide broader radiation coverage than highly heterogeneous tumor-cell targets. The expression of FAP across multiple epithelial tumors further supports this strategy in GEP malignancies. 16 21

Clinical FAPI PET studies have shown high uptake in several GEP malignancies, particularly pancreatic and biliary cancers, with favorable tumor-to-background contrast in the abdomen. These findings support the in vivo accessibility of FAP-positive stroma and provide the basis for a theranostic approach using diagnostic FAPI imaging and therapeutic ^177^ Lu-labeled analogues. However, uptake heterogeneity between and within tumors supports the need for imaging-based patient selection. 22 23 Although early FAPI tracers showed rapid tumor uptake, their relatively fast washout limited their therapeutic applicability. This has driven the development of modified ligands with improved tumor retention, including peptide-based and other retention-enhanced constructs. Early clinical experience suggests that ^177^ Lu-FAP–based therapy is feasible and generally tolerable, although current evidence remains preliminary and derives mainly from small cohorts. 24 25

### The FAPI Theranostic Concept: From Imaging to Therapy

FAPI PET/CT enables in vivo characterization of the tumor stromal compartment by targeting FAP expression on activated CAFs, reflecting tumor microenvironment remodeling rather than tumor-cell metabolism or lineage-specific receptor expression. 26 In abdominal oncology, its practical advantage lies in generally low physiologic background in many normal tissues, which may improve detection of peritoneal, nodal, and soft-tissue disease. This favorable biodistribution and imaging contrast were demonstrated in early human biodistribution and dosimetry studies using DOTA-containing FAPI agents, providing a biologic and technical rationale for imaging in complex GEP anatomy. 23

In this review, terminology related to radionuclide therapy follows the recently proposed international consensus nomenclature, which recommends the use of “radioligand therapy (RLT)” as a standardized term to describe targeted radionuclide delivery using receptor- or ligand-based approaches. 6 Because FAP-targeted RLT delivers radiation to a predominantly stromal target, baseline target expression and in vivo uptake heterogeneity are key determinants of absorbed dose distribution and, consequently, clinical benefit. Early clinical FAPI PET studies across different cancers showed substantial variation in uptake between tumor types and lesions, supporting biomarker-guided selection as a fundamental requirement for therapeutic application. 27

Unlike somatostatin receptor imaging or PSMA PET, where semiquantitative scoring systems such as the modified Krenning score and miPSMA score support treatment eligibility, 28 29 FAP-targeted RLT lacks a standardized visual or quantitative framework. Current practice relies on pragmatic PET-based criteria, including uptake greater than that of reference tissues such as the blood pool or liver, or semiquantitative thresholds (e.g., SUV or tumor-to-background ratio). 30 These approaches emphasize not only uptake intensity but also lesion homogeneity and background activity, all of which influence the therapeutic index.

A key distinction between FAPI imaging and FAPI theranostics is the need to translate tracer uptake into time-integrated activity and residence time, and subsequently into absorbed dose for both tumors and organs at risk. Early human biodistribution studies of DOTA-containing FAPI tracers provided preliminary dosimetry data and showed that imaging-favorable kinetics, such as rapid clearance and high contrast, do not necessarily translate into therapy-favorable kinetics characterized by sustained tumor retention. 23

This limitation has driven ligand engineering as a central component of therapeutic development. Although early FAPI inhibitor research anticipated labeling with both 68Ga for imaging and therapeutic radionuclides such as ^177^ Lu, subsequent development has increasingly focused on constructs designed to enhance tumor retention, including peptide-based and other retention-optimized ligands. Thus, theranostic translation is not simply a matter of replacing 68Ga with ^177^ Lu, but of optimizing pharmacokinetics (PK) to preserve target binding while prolonging intratumoral residence time and absorbed dose delivery. 31

Early therapeutic experience with peptide-based ^177^ Lu-FAP agents, particularly ^177^ Lu-FAP-2286, has illustrated this clinical workflow: baseline target imaging is used for eligibility, therapy is delivered in cycles, and post-therapy or dosimetry assessments are used to estimate organ doses, including those to the kidneys and marrow, and to evaluate feasibility. Although current datasets remain early-phase and heterogeneous, they establish the practical framework of FAPI theranostics: selection, treatment, dosimetry and safety monitoring, and response assessment. 32

A major limitation in GEP-focused practice is the lack of harmonized reporting linking baseline PET parameters, tumor absorbed dose, and clinical outcomes. Future studies should prospectively define PET-based selection thresholds, clarify how benign background uptake is addressed, standardize dosimetry methodology and imaging time points, and report lesion-level response alongside tumor dose, as stromal targeting makes the relationship between uptake and tumor control particularly dependent on spatial distribution and PK.

### Radiopharmaceutical Landscape for ^177^ Lu-FAP Therapy

^177^ Lu-FAP therapy should be regarded as a group of radioligands that share the same target, FAP, but differ in pharmacophore class, chelator and linker design, multimerization, and PK optimization. These structural differences substantially influence tumor retention, normal-organ exposure, particularly in the kidneys, liver, and bowel, and ultimately the therapeutic index ( Table 1 ). 33 34

#### Quinoline Small-Molecule FAPI Derivatives (The “FAPI-04/46 Lineage”)

Clinically, quinoline-based tracers such as FAPI-04 and FAPI-46 established the feasibility of FAP targeting by demonstrating high tumor uptake across multiple epithelial malignancies, including gastrointestinal cancers, and they formed the imaging basis for patient selection in therapeutic studies. 35 Early clinical dosimetry studies with 68Ga-FAPI agents showed favorable diagnostic biodistribution and imaging kinetics, but also identified a major therapeutic limitation of many small-molecule constructs: relatively rapid washout from tumor lesions. 36

#### DOTA/DOTAGA “SA.FAPi” Constructs and Dimerization (Retention-Focused Chemistry)

An important medicinal chemistry strategy has been multimerization or dimerization, as in DOTAGA.(SA.FAPi)_2, to prolong lesion retention and increase absorbed tumor dose. This benefit, however, may be accompanied by higher physiologic retention in hepatobiliary and gastrointestinal tissues in some designs. 37

Early clinical experience with ^177^ Lu-DOTAGA.(SA.FAPi)_2 and related constructs helped define organ dosimetry and tumor retention patterns in advanced cancers, supporting the concept that structural modification, including dimerization, can meaningfully improve lesion residence time. 38 A more recent example is ^177^ Lu-DOTAGA.Glu.(FAPi)_2, which reflects the transition from proof-of-concept development to cohort-level clinical evaluation in defined malignancies such as sarcoma. 39

#### Peptide Ligands: FAP-2286 (A Distinct Binding/PK Phenotype)

FAP-2286 represents a peptide-based strategy that is mechanistically and pharmacokinetically distinct from earlier quinoline small-molecule FAPI inhibitors. Unlike FAPI-04 and FAPI-46, which often show rapid tumor washout, peptide-based ligands such as FAP-2286 demonstrate more prolonged tumor retention, improving the feasibility of therapeutic radionuclide delivery. 40 This is especially important in desmoplastic tumors such as pancreatic ductal adenocarcinoma (PDAC) and cholangiocarcinoma, where strong stromal uptake alone may be insufficient without sustained ligand retention for effective dose delivery. 41

#### Albumin-Binder/Long-Acting FAPI Radioligands (Evans-Blue and Related Designs)

A parallel PK strategy has been the incorporation of albumin-binding motifs, often Evans blue-derived or related binders, to prolong blood circulation and increase tumor exposure. This approach can improve lesion retention and absorbed dose, although sometimes at the cost of greater normal-organ exposure, depending on ligand design and administered dose. 42 43

LNC1004 and ^177^ Lu-FAPI-XT exemplify newer retention-enhancing strategies developed to overcome the rapid washout and limited tumor residence time of earlier quinoline-based FAPI ligands. LNC1004 ( ^177^ Lu-EB-FAPI) uses an Evans blue–derived albumin-binding design to prolong systemic circulation and increase tumor retention, thereby improving tumor absorbed dose, though with some increase in marrow exposure. In contrast, ^177^ Lu-FAPI-XT is a long-acting FAPI construct with clinically demonstrated prolonged kinetics, including mean effective half-lives of approximately 52.1 hours for the whole body and 31.8 hours for tumor lesions in a first-in-human phase I study. Together, these agents illustrate two distinct next-generation approaches to improving radiation delivery by extending tumor residence time relative to earlier short-retention FAPI radioligands. 44 45 46

### Clinical Evidence of ^177^ Lu-FAPI RLT in GEP Malignancies

#### Pancreatic Ductal Adenocarcinoma

Among GEP malignancies, PDAC has the largest reported experience with ^177^ Lu-FAP therapy; however, the efficacy signal remains limited. Across eight patients from three reports using ^177^ Lu-FAP-46 and ^177^ Lu-FAP-2286, the predominant outcome was radiologic progression, indicating limited objective tumor control. Notably, some patients experienced meaningful symptomatic improvement, including pain relief and reduced opioid use, despite RECIST progression, suggesting a potential palliative benefit. Toxicity was generally manageable, with mild-to-moderate hematologic adverse events reported, particularly with peptide-based constructs. 32 38 47

#### Cholangiocarcinoma and Biliary Tract Cancers

Although the biological rationale for FAP-targeted therapy in biliary malignancies is strong, reflecting their stromal-rich microenvironment, dedicated clinical therapeutic data are currently lacking. Existing evidence is indirect and derived from heterogeneous cohorts rather than tumor-specific studies. This gap is clinically relevant given the frequent presence of impaired hepatic reserve and biliary complications in this population, which may affect both treatment feasibility and safety. Accordingly, current evidence remains hypothesis generating rather than definitive. 48 49 50

#### Gastric Cancers

Clinical experience in gastric cancer is limited and does not yet demonstrate a clear antitumor effect. Among four reported patients treated with ^177^ Lu-FAP-2286 or ^177^ Lu-LNC1004, radiologic progression predominated in evaluable cases, despite repeated treatment cycles. Toxicity was generally mild, and feasibility was established; however, robust efficacy signals remain absent, and site-specific outcomes for newer retention-enhanced ligands are still insufficiently defined. 44 50

#### Duodenal Adenocarcinoma

Evidence is restricted to a single reported case treated with ^177^ Lu-FAP-2286, which demonstrated disease progression despite multiple therapy cycles and acceptable tolerability. While progression-free survival was modest, this isolated observation does not support meaningful efficacy, and current evidence remains limited to feasibility and short-term safety. 50

#### Colorectal Cancer

The CRC dataset is small but suggests a modest disease-stabilization signal, particularly in colon cancer. Across reported cases treated with various FAPI constructs, stable disease was observed in a subset of patients, although objective responses remain rare. Toxicity was generally mild and manageable. In contrast, rectal cancer appears less responsive, with progression as the dominant outcome and short progression-free survival. Overall, these findings suggest limited but potentially more favorable activity in colon compared with other GEP subtypes, although this is based on small, heterogeneous, and non-comparative datasets. 32 38 44 46 50

#### Hepatocellular Carcinoma

Direct clinical evidence for ^177^ Lu-FAPI therapy in hepatocellular carcinoma (HCC) remains absent. While FAPI PET has shown diagnostic potential in liver malignancies, therapeutic data are absent. This is particularly important given the underlying liver dysfunction commonly present in HCC, which may complicate dosimetry and safety. At present, HCC should be considered an investigational indication for FAP-targeted RLT. 46 51

#### Overall Interpretation

Collectively, the current clinical literature remains in a signal-detection phase rather than efficacy confirmation. Across GEP malignancies, objective radiologic responses are rare, while disease stabilization and symptomatic benefit appear more consistent. These patterns likely reflect a combination of biological and PK limitations, including stromal targeting without direct tumor-cell binding, heterogeneous intratumoral distribution, and insufficient tumor retention with early-generation ligands. Accordingly, current evidence supports feasibility and early safety but does not yet define clear histology-specific indications, validated selection criteria, or reproducible efficacy benchmarks. 44 48 50 The clinical outcome of ^177^ Lu-FAP radioligand therapy in GEP is summarized in Table 2 .

### Safety and Dosimetry

Available human data indicate that ^177^ Lu-labeled FAP-targeted RLT has, so far, demonstrated a generally favorable early safety profile, with acceptable short-term tolerability and few severe nonhematologic toxicities. In the prospective 2025 single-center gastrointestinal cohort treated with ^177^ Lu-FAP-2286, no grade III/IV adverse events were reported across 30 treatment cycles in 14 patients, and toxicity was mainly limited to mild, transient gastrointestinal symptoms such as nausea, vomiting, and diarrhea. 50

Hematologic toxicity remains the adverse-event domain requiring the greatest caution. In the first-in-human ^177^ Lu-FAP-2286 series of 11 patients with advanced adenocarcinomas, no grade 4 toxicity occurred, but grade 3 pancytopenia and leukocytopenia were reported. In the phase II ^177^ Lu-LNC1004 trial, which evaluated a retention-enhanced Evans blue–modified construct in a broader metastatic basket including a small GEP subgroup, grade ¾ hematotoxicity occurred in 21% of patients. Collectively, these findings suggest that anemia, leukopenia, and thrombocytopenia, particularly in heavily pretreated patients, are the most plausible dose-limiting toxicities in current ^177^ Lu-FAP practice. 32 44

Renal safety appears provisionally reassuring, although current evidence is limited by small sample sizes and short follow-up. In the first-in-human ^177^ Lu-FAP-2286 study, the mean kidney absorbed dose was 1.0 ± 0.6 Gy/GBq, while no grade ¾ nephrotoxicity was reported in the ^177^ Lu-LNC1004 phase II trial. Similarly, the first-in-human dose-escalation study of ^177^ Lu-FAPI-XT reported no dose-limiting toxicity and no grade ≥3 treatment-related adverse events. These data suggest that clinically significant renal injury has not yet emerged as the dominant early toxicity signal, although confidence remains limited. 44 46

Evidence regarding hepatic toxicity is less definitive. In the ^177^ Lu-LNC1004 phase II study, no grade ¾ hepatotoxicity was observed, which is reassuring at the basket-trial level. However, these findings should not be overinterpreted for GEP malignancies, especially biliary tract cancers or HCC, because published cohorts have not specifically been enriched for patients with severe cholestasis, decompensated cirrhosis, or markedly impaired liver reserve. Thus, major hepatotoxicity has not emerged as a prominent early signal, but hepatic safety remains insufficiently defined in the most vulnerable subgroups. 44

Other adverse effects have generally been mild and nonspecific, including fatigue, transient gastrointestinal symptoms, and occasional pain flare. In the first-in-human ^177^ Lu-FAP-2286 report, pain response was observed in some patients, although one grade 3 pain flare-up was also documented. 32 50

#### Dosimetry Insights

Across the available ^177^ Lu-FAP therapeutic literature, dosimetry consistently identifies the kidneys and bone marrow as the key organs at risk, although the clinically dose-limiting compartment appears to depend on ligand architecture, tumor burden, prior treatment exposure, and tracer retention kinetics. In the first-in-human ^177^ Lu-FAP-2286 study, mean absorbed doses were 1.0 ± 0.6 Gy/GBq for the kidneys and 0.05 ± 0.02 Gy/GBq for red marrow, while tumor doses were substantial but notably heterogeneous. In the phase II ^177^ Lu-LNC1004 study, the mean tumor absorbed dose was 4.69 ± 3.83 Gy/GBq, again with wide interlesional variability, and grade 3 to 4 hematotoxicity was more prominent than severe renal or hepatic toxicity. Collectively, these data suggest that although renal dosimetry remains a central consideration in FAP-targeted RLT, marrow toxicity may represent the more immediate practical constraint in heavily pretreated patients and in higher-retention therapeutic constructs. 32 44

A consistent theme in the emerging ^177^ Lu-FAP literature is the marked heterogeneity of dose delivery across both patients and lesions. In the first-in-human ^177^ Lu-FAP-2286 study, prolonged tumor retention translated into relatively high absorbed doses in selected lesions, whereas the phase II ^177^ Lu-LNC1004 study showed a wide tumor dose range of 1.18 to 25.03 Gy/GBq. In addition, the gastrointestinal ^177^ Lu-FAP-2286 cohort found no significant association between treatment efficacy and the imaging parameters assessed, indicating that pretreatment FAP PET avidity, although biologically informative, is not yet a reliable stand-alone surrogate for absorbed tumor dose or clinical response. 32 44 50

An important PK lesson is that dosimetric behavior is strongly ligand-dependent. Early experience with ^177^ Lu-FAPI-46 in metastatic PDAC showed rapid tumor washout despite favorable diagnostic uptake, underscoring that imaging suitability does not necessarily translate into adequate therapeutic residence time. By contrast, retention-enhanced constructs, including the peptide-based ^177^ Lu-FAP-2286, the Evans blue–modified ^177^ Lu-LNC1004, and ^177^ Lu-FAPI-XT, have demonstrated more prolonged tumor retention and more favorable tumor dose delivery in early clinical studies. Accordingly, ^177^ Lu-FAP RLT should be considered a ligand-dependent platform rather than a dosimetrically uniform class effect. 32 44 46

#### Practical Safety Recommendations (Evidence-Informed, Cautious)

In early clinical use of ^177^ Lu-FAP RLT, particular caution is warranted in patients with limited hematologic reserve, impaired renal function, or clinically significant hepatic dysfunction. Although formal exclusion criteria specific to GEP malignancies have not yet been established, this conservative approach is supported by the current safety and dosimetry literature. Hematologic toxicity has emerged as the most clinically relevant serious adverse-event signal, while renal exposure remains a consistent dosimetric concern. Likewise, eligibility criteria in the phase II ^177^ Lu-LNC1004 study and the phase I ^177^ Lu-FAPI-XT trial emphasized preserved marrow, renal, and hepatic function together with acceptable performance status, supporting a cautious organ function-based selection strategy. 44 46

In the absence of a validated ^177^ Lu-FAP–specific surveillance protocol, a reasonable monitoring strategy would include baseline assessment of complete blood count, renal function, liver biochemistry, albumin, and performance status before each treatment cycle, followed by early post-therapy laboratory reassessment within approximately 1 to 2 weeks and repeat evaluation before subsequent administration. This should be regarded as an evidence-informed extrapolation from current ^177^ Lu-FAP studies and broader RLT practice rather than a formal standard. Such monitoring is particularly relevant in heavily pretreated patients, those with diffuse osseous or marrow involvement, and those receiving retention-enhanced ligands. 32 44 52

The most defensible overall conclusion is that short-term severe nonhematologic toxicity has been uncommon, the kidneys remain consistently relevant in dosimetry but have not yet emerged as the dominant clinical toxicity site, and marrow suppression is the adverse-event domain that most warrants caution. In contrast, the long-term renal safety ceiling, the safety of treatment in decompensated hepatobiliary disease, and reliable dose thresholds predicting benefit in GEP malignancies remain undefined and should be explicitly recognized as major evidence gaps.

### Response Assessment Challenges in FAP-Targeted RLT

In the current human ^177^ Lu-FAP literature, response assessment is still largely adapted from conventional oncologic frameworks rather than designed specifically for stromal-targeted RLT. The most commonly reported endpoints include morphologic response by Response Evaluation Criteria in Solid Tumors (RECIST) 1.1, metabolic response using PET Response Evaluation Criteria in Solid Tumors (PERCIST)-based PET criteria, and broader measures of clinical benefit such as symptom improvement or changes in performance status. This pattern is illustrated in the prospective gastrointestinal ^177^ Lu-FAP-2286 study, in which both RECIST 1.1 and PERCIST 1.0 were used, reflecting an emerging practice of combining anatomical and metabolic assessment in GEP malignancies. 50 53 54

A similar approach is emerging beyond GEP disease. In a 2026 retrospective study of ^177^ Lu-FAP-2286 for pleural metastases, response was assessed using FAP PET/CT, CT, and qualitative post-therapy FAP SPECT/CT, with high concordance reported between FAP PERCIST 1.0 and RECIST 1.1. Methodologically, this suggests movement toward a FAP-adapted molecular response framework. 54 55

Biochemical markers are sometimes reported in parallel, particularly in GEP malignancies where carbohydrate antigen 19–9 (CA19–9) and carcinoembryonic antigen (CEA) are routinely used, but they are problematic as stand-alone endpoints for FAP RLT. CA19–9 can be affected by biliary obstruction, cholangitis, and pancreatitis, while CEA has limited sensitivity and specificity. These markers may provide supportive clinical information, but they should not be regarded as direct surrogates of radioligand response without imaging correlation. 56 57 58

#### Why Standard Criteria May Be Suboptimal

Because FAP-targeted RLT acts primarily on the tumor stroma rather than directly on malignant cells, early treatment effects may manifest as reduced stromal activity, extracellular matrix remodeling, or disruption of CAF-mediated support before any measurable shrinkage appears on CT. This is particularly plausible in desmoplastic GEP malignancies such as PDAC and biliary cancers, where reliance on RECIST alone may underestimate early pharmacodynamic benefit. 53 At the same time, molecular response assessment with FAPI PET also remains insufficiently defined: unlike FDG PET, FAPI imaging reflects stromal biology rather than glucose metabolism, so changes in tracer uptake may indicate true response, inflammatory remodeling, treatment-related stromal perturbation, or lesion-level heterogeneity. Accordingly, validated thresholds for meaningful metabolic response in GEP FAP RLT are still lacking, and current interpretations remain largely hypothesis-generating rather than evidence-based. 50 54

Taken together, these limitations support a cautious and composite approach to response assessment. Apparent early progression or fluctuating imaging findings may not always represent true treatment failure, especially in future combination strategies involving immunotherapy, external-beam radiation, or other microenvironment-modifying interventions; in this context, iRECIST remains conceptually relevant as a reminder that atypical response kinetics may require contextual interpretation rather than immediate classification as progression. 53 59 Therefore, the most defensible framework for evaluating FAP-targeted RLT in GEP malignancies is an integrated model combining anatomical imaging, molecular imaging, symptom burden, and disease-specific biomarkers, particularly since early studies suggest that baseline imaging parameters do not consistently correlate with clinical benefit and response cannot yet be reduced to a single SUV-based metric. 55

### Where ^177^ Lu-FAPI Might Fit Clinically

Clinically, the most plausible current role of ^177^ Lu-FAPI RLT in GEP malignancies is as a biomarker-selected option for advanced, high-unmet-need disease after failure of established therapies, rather than as an early-line standard treatment. Early GI-focused data with ^177^ Lu-FAP-2286 and the phase II ^177^ Lu-LNC1004 basket study support feasibility and disease control in selected FAP-avid, heavily pretreated patients, supporting this later-line positioning. 44 46 50 Within GEP oncology, PDAC remains one of the strongest biologic candidates because of its aggressive course, chemoresistance, and highly desmoplastic CAF-rich microenvironment, although early ^177^ Lu-FAPI-46 experience suggests that high uptake alone is insufficient when tumor retention is poor; accordingly, PDAC currently appears most suitable for retention-optimized ligands and prospective trials rather than routine use. 44 47

Other potential niches remain exploratory but biologically attractive. Biliary tract cancer after systemic therapy failure is a reasonable candidate when disease is strongly FAPI-avid and other options are limited, although direct human therapeutic evidence is still minimal and safety may be complicated by liver dysfunction, cholestasis, infection, and fibrosis. 44

Peritoneal carcinomatosis, particularly from gastric, colonic, or pancreatic primaries, may represent another especially promising setting because FAPI PET appears advantageous for detecting peritoneal disease, and early case-level therapeutic experience with ^177^ Lu-FAP-2286 in gastric cancer supports this hypothesis. 60

#### Patient Selection Decision Tree

A clinically relevant decision pathway for FAP-targeted RLT may include confirmation of diagnosis and stage, baseline FAPI PET/CT for target expression and heterogeneity assessment, evaluation of marrow, renal, and hepatic reserve, ligand-specific dosimetry, and cyclic treatment with interval safety and response monitoring. This approach reflects the design of recent prospective studies, including ^177^ Lu-LNC1004, which required SUVmax ≥10 in >50% of tumors, and ^177^ Lu-FAPI-XT, which incorporated baseline 68Ga-FAPI PET/CT screening before dosimetry and therapy. 44 46

The most important practical message of that algorithm is that selection should be ligand-aware and not purely histology-driven. A patient with strong uptake but poor marrow reserve may be unsuitable despite excellent imaging, while a patient with diffuse heterogeneous uptake may be at risk for mixed response even if one dominant lesion is intensely avid. Likewise, the threshold for treatment should probably be higher in disease settings with vulnerable organ function, such as cholestatic biliary cancer or cirrhotic liver disease, because the current safety evidence is strongest in selected trial populations rather than in those fragile subgroups. 44 50

## Conclusion

^177^ Lu-FAP RLT is a biologically compelling and clinically promising strategy for advanced GEP malignancies, particularly in stromal-rich and treatment-refractory disease. Current evidence supports feasibility, manageable early toxicity, and occasional disease stabilization or symptomatic benefit, but remains too limited and heterogeneous to define clear histology-specific indications or efficacy benchmarks. Progress will depend on retention-optimized ligands, more rigorous imaging-based patient selection, standardized dosimetry and response assessment, and prospective comparative trials to clarify where FAP-targeted theranostics can deliver meaningful clinical benefit in GEP oncology.

**Table 1:** Major therapeutic FAPI platform classes and pharmacokinetic rationale

Platform class	Key examples	PK / design strategy	Dosimetric implication
**Quinoline small-molecule FAPI derivatives**	FAPI-04, FAPI-46	High-affinity small-molecule tracers mainly optimized for imaging	High tumor uptake but **rapid lesion washout** , limiting therapeutic dose delivery
**SA.FAPi dimers / multimeric constructs**	^177^ Lu-DOTAGA.(SA.FAPi) _2_ , ^177^ Lu-DOTAGA.Glu.(FAPi) _2_	Dimerization / multimerization **to** increase avidity and retention	Longer tumor residence and higher absorbed dose, with possible increased hepatobiliary retention
**Peptide ligands**	FAP-2286	Distinct peptide-based binding scaffold	Prolonged tumor retention enabling improved radionuclide dose delivery
**Albumin-binder / long-acting constructs**	LNC1004 ( ^177^ Lu-EB-FAPI), ^177^ Lu-FAPI-XT	Albumin binding or long-acting scaffold to prolong circulation	Extended systemic and tumor half-life, improving dose delivery but potentially increasing normal-organ exposure

Abbreviations: FAPI, fibroblast activation protein inhibitor; PK, pharmacokinetic.

**Table 2:** Summary of clinical outcomes and treatment characteristics of ^177^ Lu-FAP radioligand therapy across gastroenteropancreatic malignancies

Malignancy type	Studies	Patients ( *n* )	^177^ Lu-FAP construct(s)	Treatment exposure	Main response signal	Safety/follow-up
Gastric cancer	Fu et al (2025) 44 Bai et al (2025) 50	4	^177^ Lu-LNC1004; ^177^ Lu-FAP-2286	Usually two cycles; individual LNC1004 exposure not reported	Predominantly PD; no clear site-specific efficacy signal	Mostly mild toxicity; short PFS in evaluable cases
Pancreatic cancer	Baum et al (2022) 32 ; Kaghazchi et al (2022) 47 ; Baum et al (2022) 32	8	^177^ Lu-FAPI-46; ^177^ Lu-FAP-2286	Usually one to two cycles	Predominantly PD; occasional symptomatic benefit despite progression	Mostly manageable toxicity; survival data limited
Duodenal adenocarcinoma	Bai et al (2025) 50	1	^177^ Lu-FAP-2286	Three cycles	PD/PMD	Mild transient diarrhea; PFS 30 wk
Colon cancer	Assadi et al (2021) 38 ; Liu et al (2026) 46 ; Fu et al (2025) 44 ; Bai et al (2025) 50	7	^177^ Lu-FAPI-46; ^177^ Lu-FAPI-XT; ^177^ Lu-LNC1004; ^177^ Lu-FAP-2286	Usually one to four cycles	Mixed outcomes, with SD in several patients	Mostly mild toxicity; limited survival data
Rectal cancer	Baum et al (2022) 32 ; Bai et al (2025) 50	4	^177^ Lu-FAP-2286	Usually one to two cycles	Predominantly PD	Limited toxicity; short PFS

Abbreviations: PD, Parkinson disease; PFS, progression-free survival; PMD, primary movement disorder.
